# P-1613. Age, Antibody Response, and Seasonal Co-infections Shape the Risk of Symptomatic Endemic SARS-CoV-2 in Uganda

**DOI:** 10.1093/ofid/ofaf695.1791

**Published:** 2026-01-11

**Authors:** Joanne Hunt, Adam Epstein-Shuman, Justin Hardick, Annet Onzia, Rosalind Parkes-Ratanshi, Lydia Nakiyingi, Yukari C Manabe

**Affiliations:** Rollins School of Public Health, Atlanta, Georgia; National Institute of Allergy and Infectious Disease, Baltimore, Maryland; Johns Hopkins School of Medicine, Baltimore, Maryland; Infectious Disease Institute, Kampala, Kampala, Uganda; Infectious Disease Institute, Makerere University, Kampala, Kampala, Uganda; Infectious Disease Institute, Kampala, Kampala, Uganda; Johns Hopkins University School of Medicine, Baltimore, MD

## Abstract

**Background:**

COVID-19 related morbidity and mortality were lower in East Africa compared to the US despite similar exposure to SARS-CoV-2. This study examined how age, SARS-CoV-2–specific antibody responses, and co-infection with endemic coronaviruses (eCoVs) relate to the cross-sectional prevalence and symptomology of COVID-19.

**Table 1.**
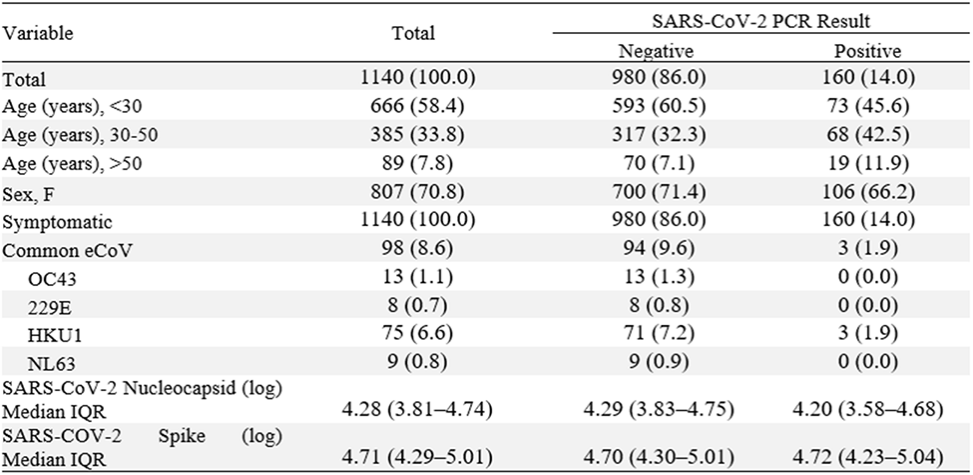
Demographics, Infection Incidence, and Clinical Features of Study Cohort. All values, unless otherwise listed, are n(%). PIV 2 and 4 were not observed and so were dropped from the table.

**Figure 1.**
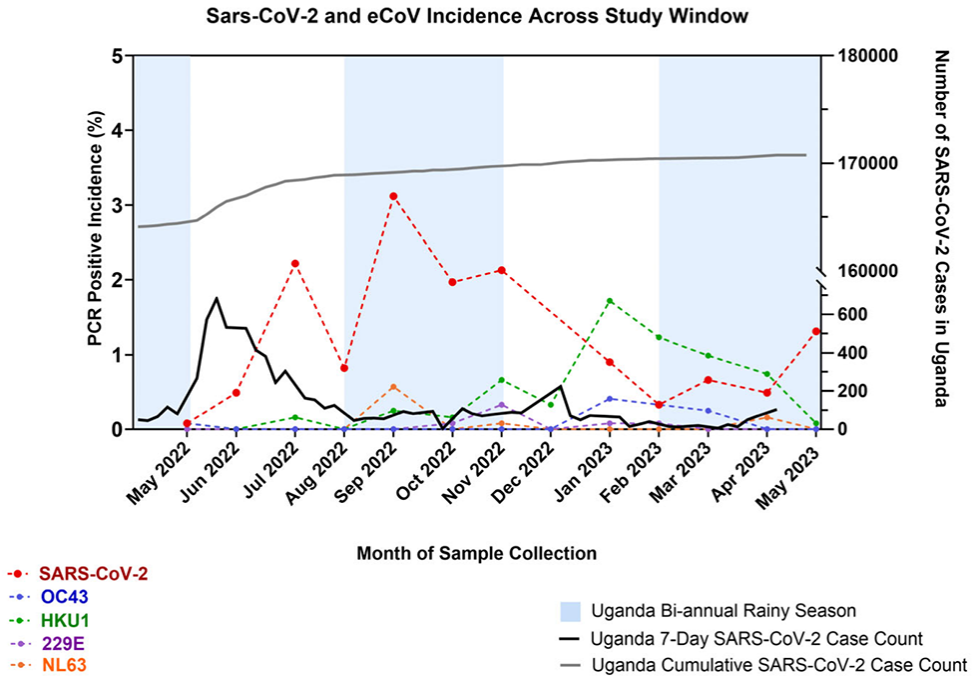
Coronavirus Incidence During Study Window In gray scale with solid lines are the Ugandan national rates of SARS-CoV-2 incidence; these correspond with the right y-axis. In color with dashed lines are the incidence of all investigated coronaviruses: SARS-CoV-2, OC43, HKU1, 229E, and NL63; these correspond with the left y-axis. The blue background panels represent the bi-annual rainy seasons in Uganda.

**Methods:**

Between May 2022 and May 2023, we recruited patients with acute respiratory symptoms from Ugandan health facilities, collecting samples and survey data. Samples underwent serologic testing and nasal swab PCR for SARS-CoV-2 and respiratory viral infections including eCoVs (229E, NL63, OC43, HKU1). We assessed monthly incidence trends using Kendall’s τ; crude odds ratios (ORs) via logistic regression and adjusted risk ratios (aRRs) via Poisson regression; and applied Spearman’s ρ and χ² tests as appropriate, applying a Benjamini–Hochberg correction.

**Figure 2.**
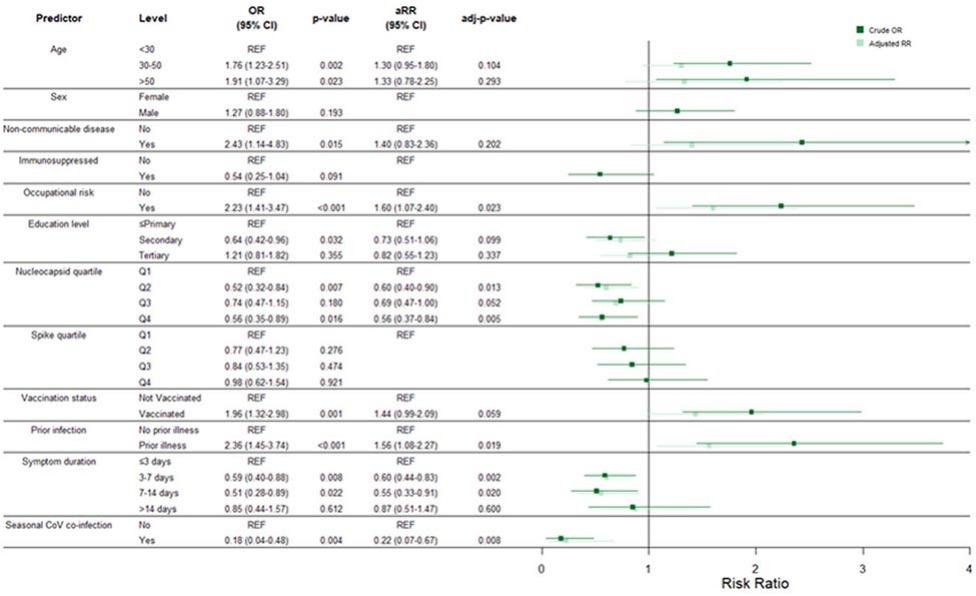
Crude and Adjusted Models Assessing Which Factors Are Linked to COVID-19 Infection.

The crude model was generated using bivariate logistic regressions. The adjusted model was created using a Poisson regression with robust standard errors. Purple represents crude while blue represents the adjusted model values. OR = odds ratio, CoV = coronavirus, REF = reference

**Results:**

Of 1140 enrolled participants (70.8% female; mean age 31 years), 14.0% tested positive for SARS-CoV-2 by PCR, 8.6% for at least one endemic coronavirus, and 1.9% for co-infection with both SARS-CoV-2 and an eCoV (Table 1). SARS-CoV-2 incidence mirrored other eCoVs (Figure 1). Occupation (aRR=1.6) and prior COVID-19 (aRR=1.6) were associated with SARS-CoV-2 infection; shorter symptom duration (< 14 days aRR≈0.6), higher anti-nucleocapsid titers (quartile aRRs≈0.6) and eCoV infection (aRR=0.2) were protective (Figure 2). Symptom burden—both count and duration—and rates of fever, hoarse voice, myalgia, and malaise—increased significantly with age, but were attenuated in the stratified analysis of SARS-CoV-2 positive participants.

**Conclusion:**

From 2022-2023, SARS-CoV-2 consistently circulated in Uganda. Exposure history increased SARS-CoV-2 risk; shorter symptom duration, robust humoral immunity and eCoV co-infection were protective. As other regions trend toward SARS-CoV-2 endemicity, understanding how pre-existing immunity and co-infection shape clinical risk can guide targeted mitigation strategies.

**Disclosures:**

Yukari C. Manabe, MD, FIDSA, FRCP, bioMerieux: Research materials to JHU|Cepheid: Research materials to JHU|Chembio: Grant/Research Support|Chembio: Research materials to JHU|Hologic: Grant/Research Support|Hologic: Research materials to JHU|Roche: Research materials to JHU

